# Analysis of odontoid parameters in adolescent idiopathic scoliosis patients with different curve types

**DOI:** 10.3389/fped.2024.1424313

**Published:** 2024-08-27

**Authors:** Kelin Li, Longao Huang, Qiongrun Xiao, Weiyou Chen, Yuwang Du, Hua Jiang

**Affiliations:** Department of Spine Surgery, The First Affiliated Hospital of Guangxi Medical University, Nanning, Guangxi, China

**Keywords:** cervical sagittal alignment, adolescent idiopathic scoliosis, odontoid incidence, odontoid parameter, thoracic curve

## Abstract

**Introduction:**

Odontoid incidence (OI) is an important parameter that has recently been developed. However, there are currently no studies on OI in adolescent idiopathic scoliosis (AIS) patients. We aimed to examine the significance of OI in describing cervical sagittal alignment in AIS patients, explore the differences in cervical sagittal parameters among these patients with different curve types, and investigate the correlations between coronal deformity and cervical sagittal parameters in AIS patients.

**Methods:**

The whole-spine anteroposterior and lateral plain radiographs of AIS patients were retrospectively analyzed. The parameters, including OI, odontoid tilt (OT), C2 slope, cervical lordosis (CL), T1 slope (T1S), and others, were measured. The AIS patients were grouped based on different curve types. Measurement parameters were compared between different groups. Pearson correlation analysis was performed for cervical sagittal parameters and Cobb angle.

**Results:**

Ninety AIS patients were included, consisting of 14 males and 76 females. The main thoracic curve group exhibited a smaller OI compared to the main thoracolumbar/lumbar curve group (*P* < 0.05). In the AIS patients with a main thoracic curve, there was a significant correlation between Cobb angle and OI (r = −0.371, *p* < 0.01). The odontoid parameters exhibited significant correlations with several classic cervical sagittal parameters in AIS patients with different curve types. The validation of the formula CL = 0.36 × OI−0.67 × OT−0.69 × T1S showed a significant correlation (correlation coefficient = 0.917) between the actual measurements and the predicted values, with a determination coefficient of 0.842.

**Conclusion:**

There may be a difference in OI between AIS patients with a main thoracic curve and those with a main thoracolumbar/lumbar curve. Odontoid parameters could be used to describe cervical sagittal alignment in AIS patients with different curve types.

## 1 Introduction

The incidence rate of adolescent idiopathic scoliosis (AIS) ranges from approximately 0.5% to 3% (1–3). AIS is a three-dimensional deformity of the spine, involving alignment abnormalities in the coronal, sagittal, and transverse planes. Currently, there is an increasing focus on sagittal plane deformities in AIS. The spinal sagittal plane is a unified whole, and any deformity in the thoracic or lumbar sagittal plane can affect the cervical spine (4–6). AIS patients have a significantly high risk of developing cervical spondylosis, which is strongly associated with cervical sagittal alignment (7, 8). Therefore, the assessment of cervical sagittal alignment is important for AIS patients.

AIS patients exhibit abnormalities in cervical sagittal alignment (9–12). Furthermore, there are significant correlations between cervical sagittal deformity and coronal deformity in AIS patients (13, 14). However, there are differences in coronal and sagittal alignment among AIS patients with different curve types (4, 15, 16). It is, therefore, necessary to conduct correlation analyses between cervical sagittal parameters and coronal deformity in AIS patients with different curve types.

Studies have been conducted on cervical sagittal alignment in various types of AIS, including C2−7 sagittal vertical axis (cSVA), cervical lordosis (CL), and T1 slope (T1S). The results thereof showed that cervical sagittal alignment is correlated with neck and shoulder pain, cervical spondylosis, and health-related quality of life (8, 17, 18). However, these parameters are not constant anatomical measurements and will change depending on the body's position. There can be variations in measurements for the same patient due to different body positions, which renders these parameters inaccurate for the assessment of interindividual differences. Odontoid incidence (OI) is an anatomical constant that remains consistent regardless of positioning and has been recently developed (19). OI can accurately describe cervical sagittal alignment (19–21). To the best of our knowledge, there are currently no reported studies on OI in AIS patients. We speculated that OI can be used to describe cervical sagittal alignment in AIS patients with different curve types.

We aimed to explore the differences in cervical sagittal parameters among AIS patients with different curve types, examine the correlations between cervical sagittal parameters and coronal plane deformities in AIS patients, and investigate the significance of OI in describing cervical sagittal alignment in AIS patients with different curve types.

## 2 Methods

### 2.1 Participants

After obtaining consent from the Institutional Review Board at the First Affiliated Hospital of Guangxi Medical University (2024-E057-01), a retrospective analysis was conducted on clinical and radiological data. The imaging data of AIS patients treated at this hospital between June 2018 and October 2023 were analyzed and collected. The inclusion criteria were as follows: (1) patients with AIS aged 10 to 18 years; (2) with whole-spine anteroposterior and lateral plain radiographs that were complete and clear. The exclusion criteria were as follows: (1) a history of spinal surgery or trauma; (2) spinal congenital malformation, suppurative spondylitis, spinal tuberculosis, spinal tumor, or ankylosing spondylitis because these conditions could alter the spinal parameters. Based on these criteria, the data from a total of 90 patients were collected.

The AIS patients were divided into two groups based on the main thoracic curve and the main thoracolumbar/lumbar curve. The types of scoliosis were identified according to Lenke classification (22). The measurement parameters were compared between the two groups. Pearson correlation analysis was conducted on cervical parameters and Cobb angle.

### 2.2 Acquisition conditions

The radiographic imaging protocol for full-spine anteroposterior and lateral views was standardized. Each patient was asked to stand upright with proper posture, place their feet naturally apart, distribute their body weight evenly between both legs without leaning toward either leg, relax the shoulders, and keep their eyes looking straight ahead.

### 2.3 Measurement of parameters

**Figure 1:**
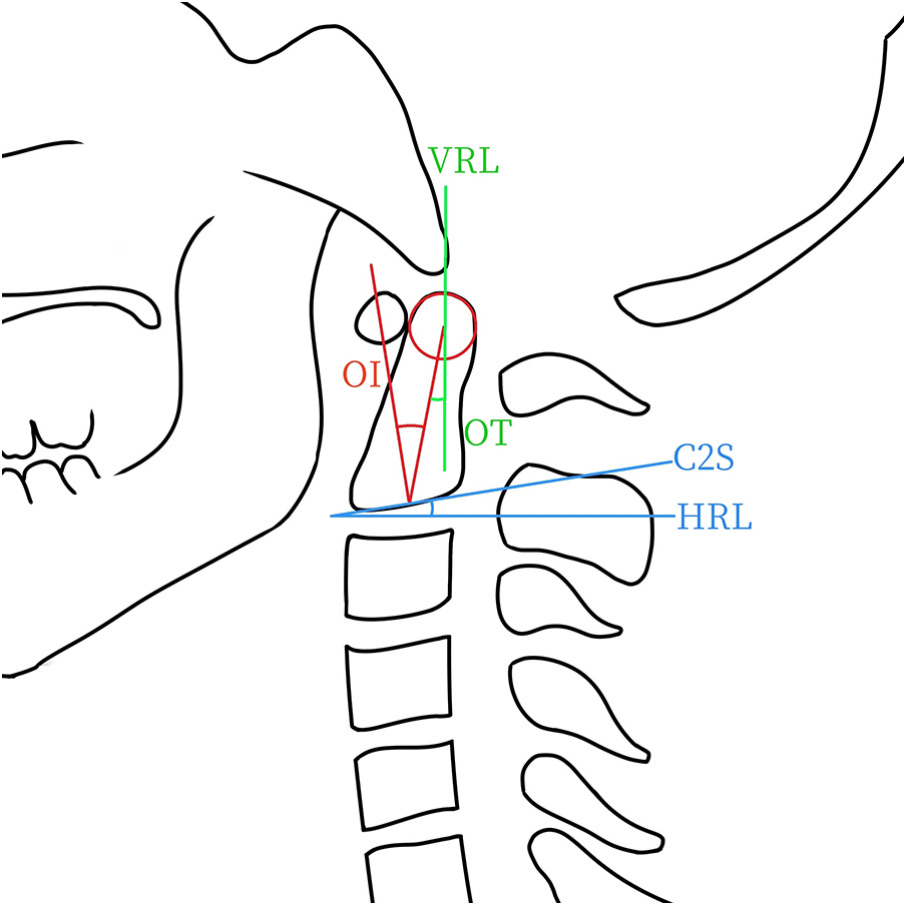
This schematic diagram illustrates the measurements of odontoid parameters, including odontoid incidence (OI), odontoid tilt (OT), and C2 slope (C2S). Geometrically, OI equals OT plus C2S. HRL, horizontal line. VRL, vertical line.

OI was defined as the angle between the line connecting the midpoint of the inferior endplate line of C2 to the odontoid center (the center of the circle tangentially touches the anterior side, posterior side, and tip of the odontoid), and a perpendicular line drawn from the inferior endplate line of C2. The odontoid tilt (OT) was defined as the angle between the line connecting the midpoint of the inferior endplate line of C2 to the odontoid center and the plumb line (19). The C2 slope (C2S) was defined as the angle between the lower endplate line of C2 and a horizontal line (Figure 1).

**Figure 2:**
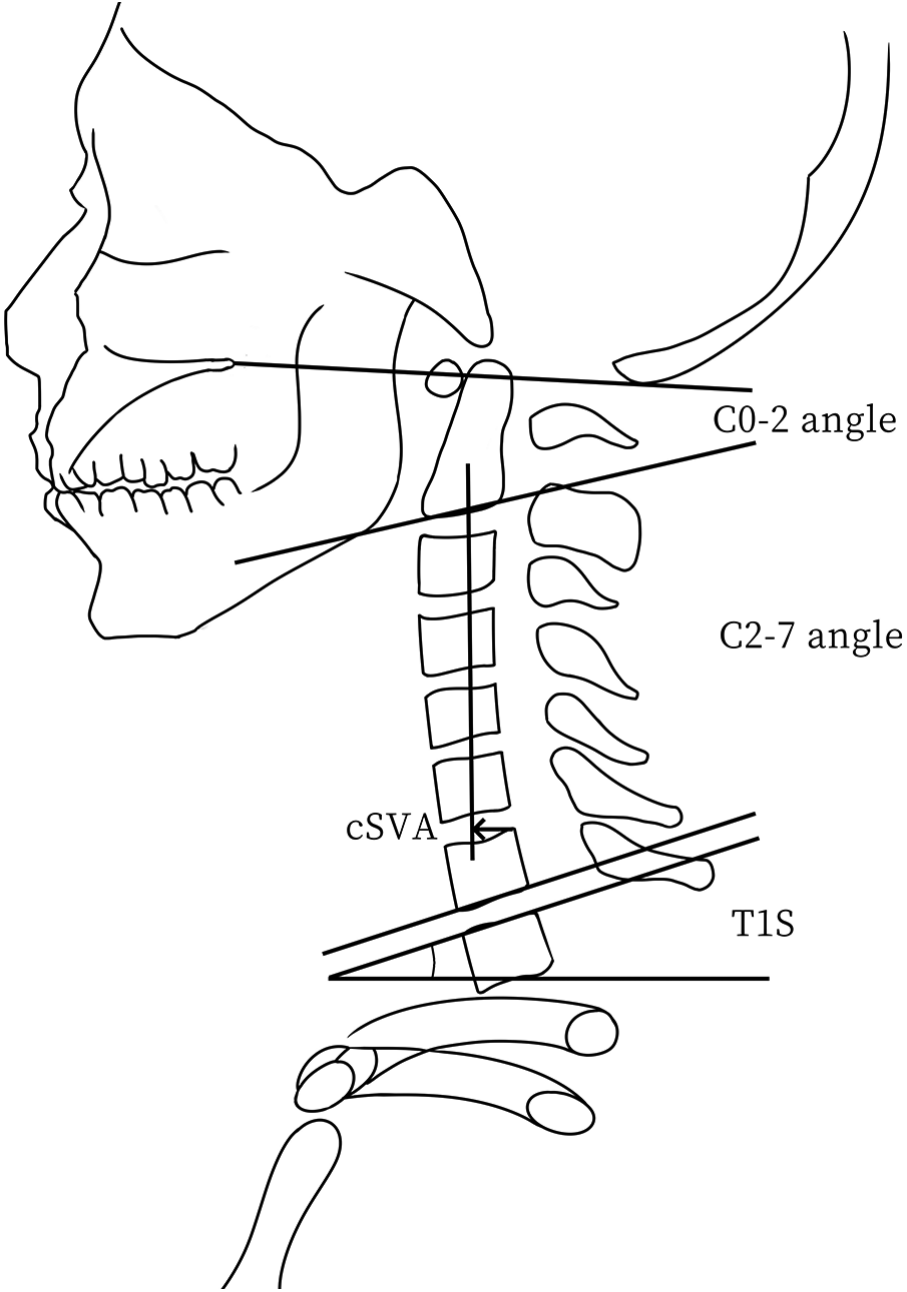
This schematic diagram illustrates the measurements of key cervical sagittal parameters, including the C2−C7 sagittal vertical axis (cSVA), T1 slope (T1S), C0−2 angle, and C2−7 angle.

**Figure 3:**
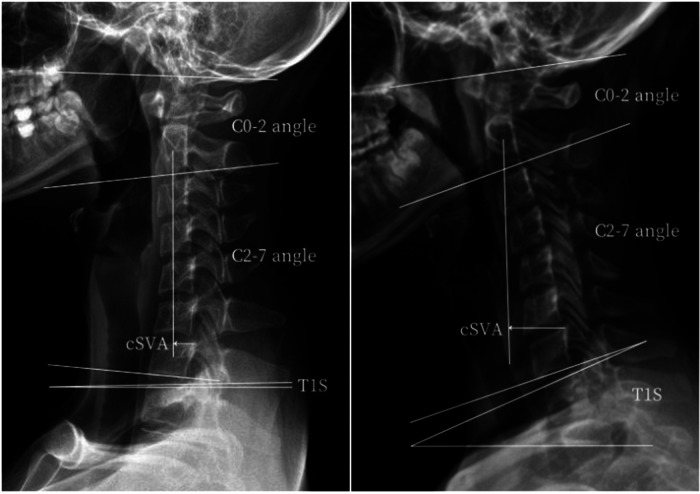
Measurements of the key cervical sagittal parameters in AIS patients with a main thoracic curve (left), or with a main thoracolumbar/lumbar curve (right).

The C0−2 angle was defined as the angle between McGregor's line and the lower endplate line of C2. The C2−7 angle was defined as the angle between the inferior endplate line of C2 and the inferior endplate line of C7. The C2−7 angle also refers to the CL. The T1S refers to the angle between the upper endplate line of T1 and a horizontal line. The cSVA was defined as the horizontal distance from the posterior superior edge of C7 to the vertical line passing through the center of C2 (Figures 2, 3). Thoracic kyphosis (TK) refers to the angle between the upper endplate line of T4 and the lower endplate line of T12. Lumbar lordosis (LL) refers to the angle between the upper endplate line of L1 and the upper endplate line of S1. The pelvic incidence (PI) was defined as the angle between the line perpendicular to the superior endplate line of S1, and the line connecting the midpoint of the bilateral femoral head centers with the midpoint of the superior endplate of S1. Pelvic tilt (PT) refers to the angle between the plumb line and the line connecting the midpoint of the bilateral femoral head centers to the midpoint of the superior endplate line of S1. The sacral slope (SS) is the angle between the endplate line of S1 and a horizontal line. The sagittal vertical axis (SVA) refers to the horizontal distance from the upper posterior edge of S1 to the vertical line that passes through the center of C7.

Positive numbers were used to record lordosis, while negative numbers were used for kyphosis. Spinal parameters were measured using the professional spine measurement software Surgimap (Nemaris, Inc., New York, NY, USA). All parameters were measured by two senior spinal surgeons, and the final statistical analysis was based on the average of their measurements.

### 2.4 Statistical analysis

SPSS (version 26) was utilized to conduct the statistical analysis. The intraclass correlation coefficient (ICC) was used to analyze the inter-observer reliability (23). The range of ICC values is from 0 to 1, with a higher value indicating greater consistency. The suggestion of Landis (24) states that ICC values between 0 and 0.4 indicate poor consistency, whereas those between 0.4 and 0.74 indicate good consistency, and those between 0.75 and 1 indicate excellent consistency. The Shapiro‒Wilk test was used to test the normality of the distribution. The parameters between two groups were compared using the independent samples *t*-test. When the data did not conform to a normal distribution, the rank-sum test was used. Using Pearson correlation analysis, the correlations among the cervical sagittal parameters were examined. A *p*-value < 0.05 indicated statistical significance.

## 3 Results

### 3.1 Inter-observer reliability

The ICC values for the measured parameters were as follows: 0.982 (Cobb angle), 0.930 (OI), 0.963 (OT), 0.954 (C2S), 0.901 (C0‒2 angle), 0.959 (CL), 0.917 (T1S), 0.864 (T1S-CL), 0.928 (TK), 0.910 (LL), 0.920 (PI), 0.867 (PT), 0.935 (SS), 0.886 (cSVA), and 0.913 (SVA). All ICC values for the parameters were >0.75, indicating excellent consistency between observers.

### 3.2 Comparison of parameters

A total of 90 patients with AIS were included, consisting of 14 males and 76 females. All measurements of the parameters and subject demographics are shown in Table 1. The AIS patients were divided into two groups according to different curve types. Compared to the main thoracic curve group, the main thoracolumbar/lumbar curve group demonstrated a higher mean OI, while showing a lower mean Cobb angle (Table 2).

**Table 1:** Parameters and demographic information of AIS patients (14 males and 76 females).

Parameters	Mean ± standard deviation (minimum—maximum)
Age(yr)	14.4 ± 2.0 (10.0 to 18.0)
Cobb(°)	45.4 ± 15.9 (20.1 to 101.7)
OI(°)	17.3 ± 4.2 (8.5 to 35.1)
OT(°)	5.5 ± 8.8 (−13.9 to 28.2)
C2S(°)	11.8 ± 8.6 (−13.0 to 33.8)
C0-2(°)	17.9 ± 8.6 (−10.8 to 44.3)
CL(°)	2.2 ± 13.4 (−40.5 to 40.3)
T1S(°)	15.6 ± 13.4 (−9.5 to 37.5)
T1S-CL(°)	13.3 ± 9.1 (−23.4 to 37.4)
TK(°)	−19.1 ± 13.1 (−45.1 to 21.3)
LL(°)	41.8 ± 12.8 (−3.3 to 70.1)
PI(°)	46.4 ± 13.7 (15.7 to 80.4)
PT(°)	10.0 ± 13.7 (−11.4 to 27.0)
SS(°)	36.4 ± 9.6 (10.9 to 58.7)
cSVA(cm)	1.3 ± 0.8 (−0.1 to 3.6)
SVA(cm)	0.6 ± 3.1 (−7.2 to 15.1)

OI, odontoid incidence; OT, odontoid tilt; C2S, C2 slope; CL, cervical lordosis; T1S, T1 slope; TK, thoracic kyphosis; LL, lumbar lordosis; PI, pelvic incidence; PT, pelvic tilt; SS, sacrum slope; cSVA, C2-C7 sagittal vertical axis; SVA, sagittal vertical axis.

**Table 2:** AIS patients were divided into group A and group B based on the type of scoliosis.

Variable	Mean ± standard deviation			*P*-value
	Group A (main thoracic curve)		Group B (main thoracolumbar /lumbar curve)	
Age(yr)	14.1 ± 2.0		14.8 ± 2.0	0.113
Cobb(°)	48.8 ± 16.7		40.4 ± 13.3	**0.018 < 0.05**
OI(°)	16.4 ± 4.5		18.5 ± 3.5	**0.010 < 0.05**
OT(°)	5.6 ± 8.8		5.2 ± 9.0	0.830
C2S(°)	10.8 ± 9.1		13.3 ± 7.8	0.177
C0-2(°)	17.4 ± 11.8		18.5 ± 8.0	0.631
CL(°)	2.4 ± 14.8		2.1 ± 11.2	0.974
T1S(°)	14.2 ± 9.2		17.5 ± 7.0	0.069
T1S-CL(°)	11.9 ± 10.1		15.4 ± 7.2	0.067
TK(°)	−18.4 ± 13.0		−20.0 ± 13.4	0.570
LL(°)	41.0 ± 14.8		42.9 ± 9.2	0.505
PI(°)	45.3 ± 14.4		48.2 ± 12.7	0.325
PT(°)	9.2 ± 8.5		11.1 ± 8.8	0.314
SS(°)	36.0 ± 10.5		37.0 ± 8.2	0.623
cSVA(cm)	1.3 ± 0.8		1.2 ± 0.7	0.961
SVA(cm)	0.7 ± 3.6		0.5 ± 2.3	0.706

OI, odontoid incidence; OT, odontoid tilt; C2S, C2 slope; CL, cervical lordosis; T1S, T1 slope; TK, thoracic kyphosis; LL, lumbar lordosis; PI, pelvic incidence; PT, pelvic tilt; SS, sacrum slope; cSVA, C2-C7 sagittal vertical axis; SVA, sagittal vertical axis.

The *P*-values of OI and Cobb angle were lower than 0.05.

### 3.3 Pearson correlation analysis

**Figure 4:**
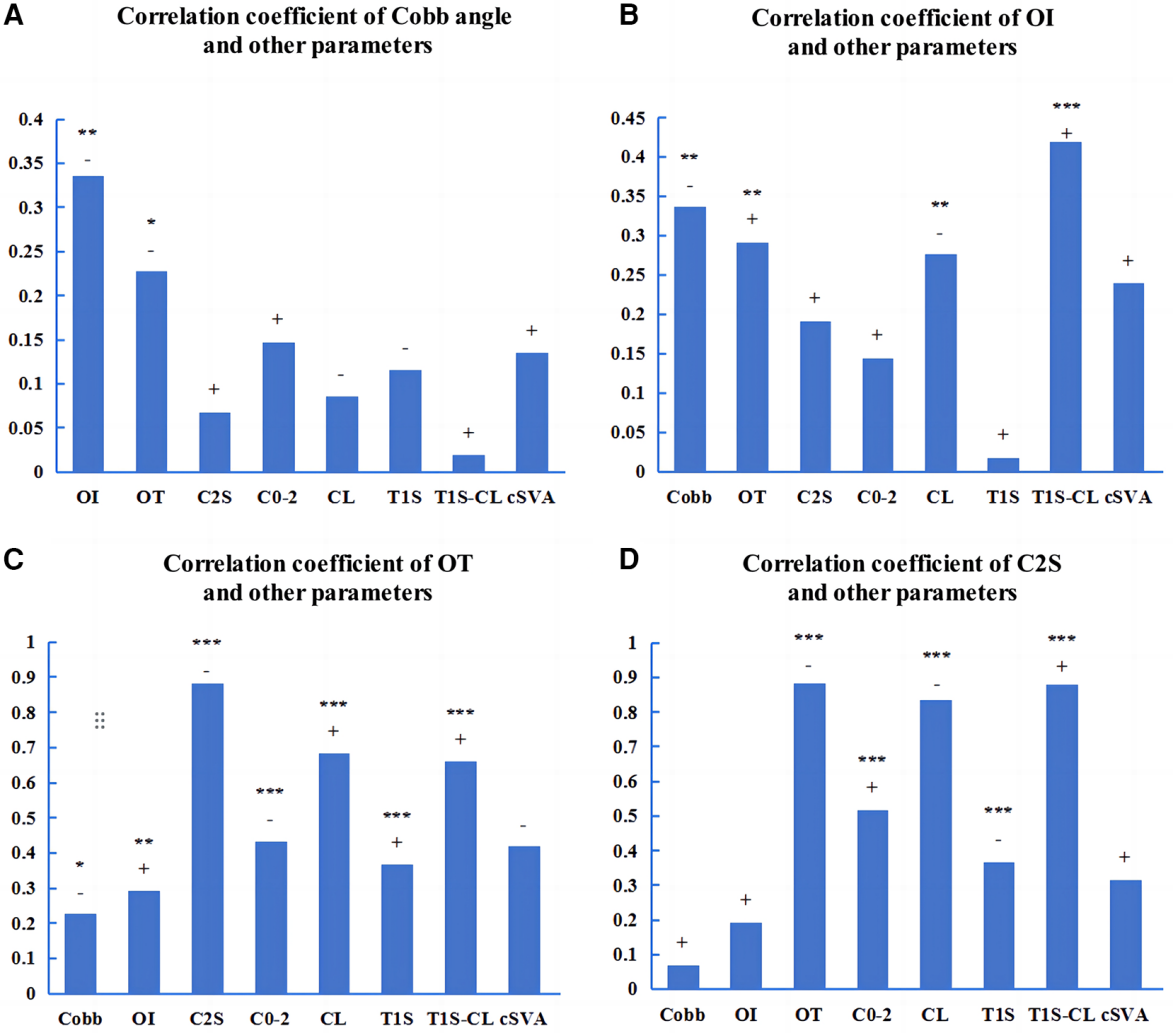
Pearson correlations among the parameters of AIS patients. **(A−D)**: The correlation coefficients among the parameters. OI, odontoid incidence. OT, odontoid tilt. C2S, C2 slope. “+” or “−” represents a positive or negative correlation. *p < 0.05. **p < 0.01. ***p < 0.001.

In all AIS patients, there were significant correlations between Cobb angle and OI (r = −0.336, *p* < 0.01) or OT (r = −0.227, *p* < 0.05) (Figure 4A). The OI showed significant correlations with several cervical sagittal parameters, including OT (r = 0.291, *p* < 0.01), CL (r = −0.276, *p* < 0.01), T1S-CL (r = 0.419, *p* < 0.001), and cSVA (r = −0.239, *p* < 0.05) (Figure 4B). The OT showed significant correlations with several cervical sagittal parameters, including C2S (r = −0.883, *p* < 0.001), C0−2 angle (r = −0.434, *p* < 0.001), CL (r = 0.682, *p* < 0.001), T1S (r = 0.366, *p* < 0.001), T1S-CL (r = 0.659, *p* < 0.001), and cSVA (r = −0.419, *p* < 0.001) (Figure 4C). C2S showed significant correlations with several cervical sagittal parameters, including the C0−2 angle (r = 0.516, *p* < 0.001), CL (r = −0.835, *p* < 0.001), T1S (r = −0.367, *p* < 0.001), T1S-CL (r = 0.881, *p* < 0.001), and cSVA (r = 0.314, *p* < 0.01) (Figure 4D).

**Figure 5:**
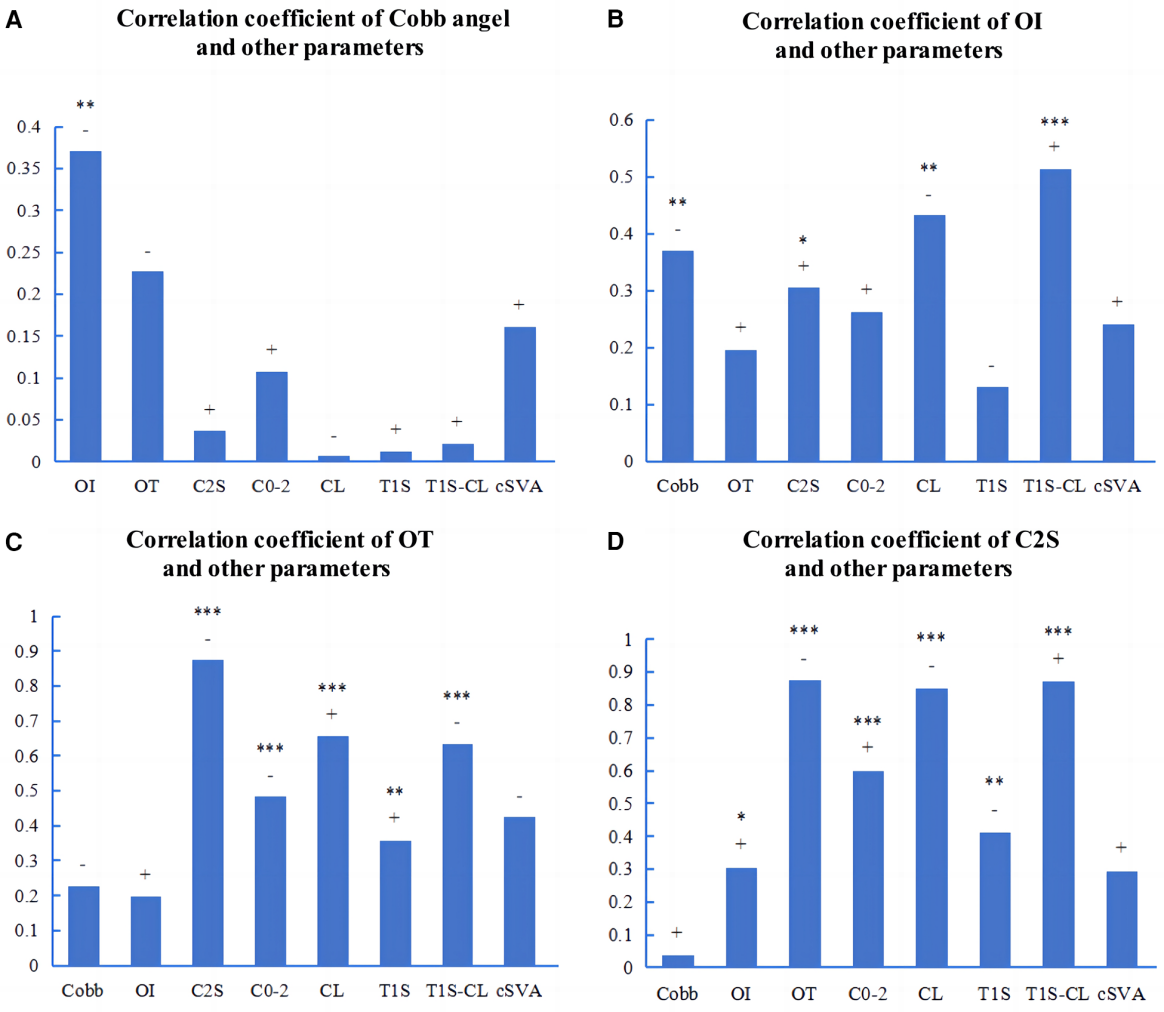
Pearson correlations among the parameters of AIS patients with a main thoracic curve. **(A−D)**: The correlation coefficients among the parameters. OI, odontoid incidence. OT, odontoid tilt. C2S, C2 slope. “+” or “−” represents a positive or negative correlation. *p < 0.05. **p < 0.01. ***p < 0.001.

In the AIS patients with a main thoracic curve, there was a significant correlation between Cobb angle and OI (r = −0.371, *p* < 0.01) (Figure 5A). The OI showed significant correlations with several cervical sagittal parameters, including C2S (r = 0.305, *p* < 0.05), CL (r = −0.433, *p* < 0.01), and T1S-CL (r = 0.514, *p* < 0.001) (Figure 5B). The OT exhibited significant correlations with several cervical sagittal parameters, including C2S (r = −0.874, *p* < 0.001), C0−2 angle (r = −0.483, *p* < 0.001), CL (r = 0.656, *p* < 0.001), T1S (r = 0.358, *p* < 0.01), T1S-CL (r = 0.634, *p* < 0.001), and cSVA (r = −0.424, *p* < 0.01) (Figure 5C). The C2S showed significant correlations with several cervical sagittal parameters, including the C0−2 angle (r = 0.599, *p* < 0.001), CL (r = −0.851, *p* < 0.001), T1S (r = −0.413, *p* < 0.01), T1S-CL (r = 0.870, *p* < 0.001), and cSVA (r = 0.293, *p* < 0.05) (Figure 5D).

**Figure 6:**
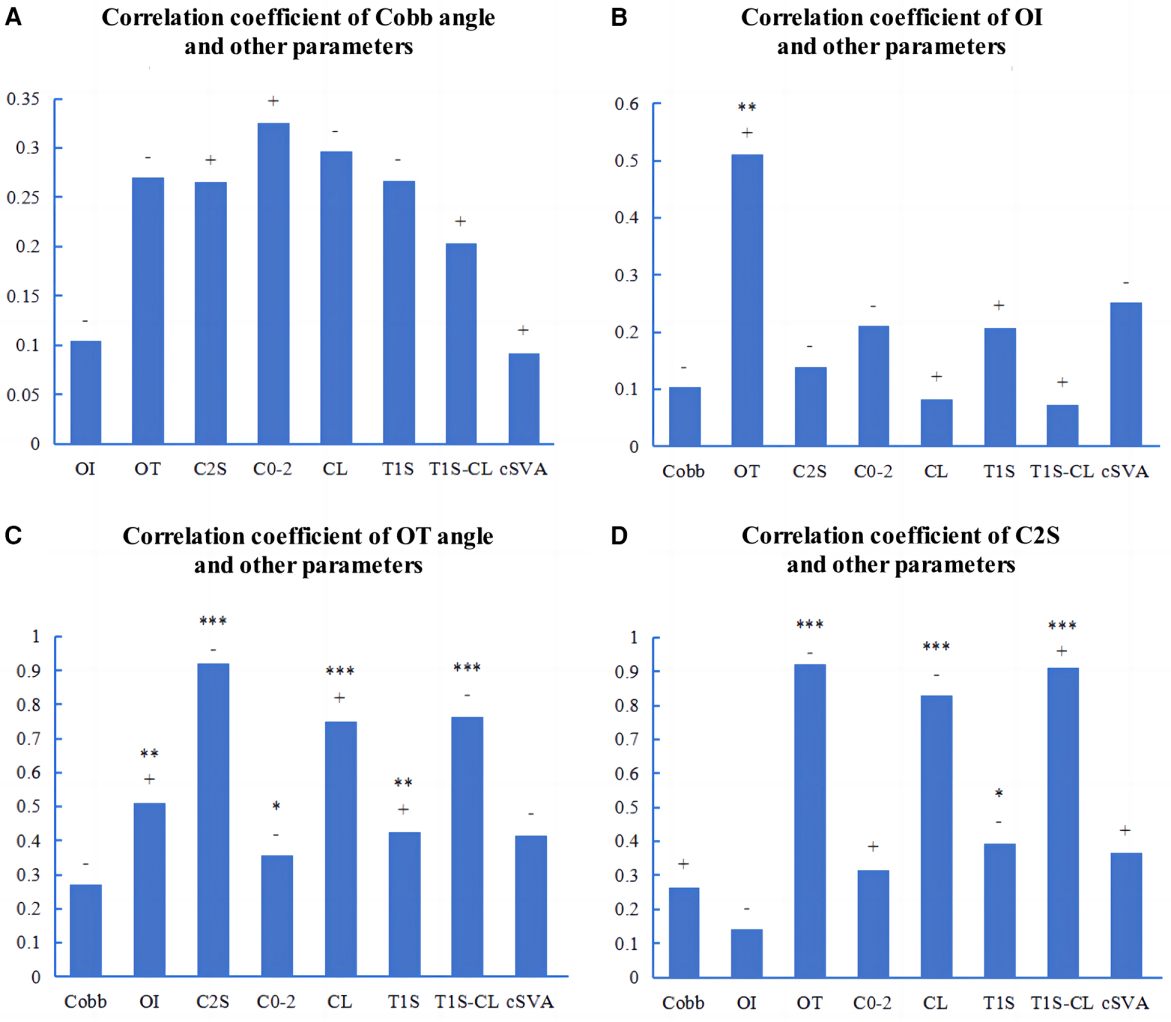
Pearson correlations among the parameters of AIS patients with a main thoracolumbar/lumbar curve. **(A−D)**: The correlation coefficients among the parameters. OI, odontoid incidence. OT, odontoid tilt. C2S, C2 slope. “+” or “−” represents a positive or negative correlation. *p < 0.05. **p < 0.01. ***p < 0.001.

In the AIS patients with a main thoracolumbar/lumbar curve, there was no significant correlation between the cobb angle and cervical sagittal parameters (Figure 6A). There was a significant correlation between OI and OT (r = 0.511, *p* < 0.01) (Figure 6B). The OT showed significant correlations with several cervical sagittal parameters, including C2S (r = −0.922, *p* < 0.001), C0−2 angle (r = −0.356, *p* < 0.05), CL (r = 0.751, *p* < 0.001), T1S (r = 0.424, *p* < 0.01), T1S-CL (r = −0.764, *p* < 0.001), and cSVA (r = −0.415, *p* < 0.05) (Figure 6C). The C2S exhibited significant correlations with several cervical sagittal parameters, including the CL (r = −0.828, *p* < 0.001), T1S (r = −0.395, *p* < 0.05), T1S-CL (r = 0.913, *p* < 0.001), and cSVA (r = 0.365, *p* < 0.05) (Figure 6D).

### 3.4 Validation of formula efficacy

**Figure 7:**
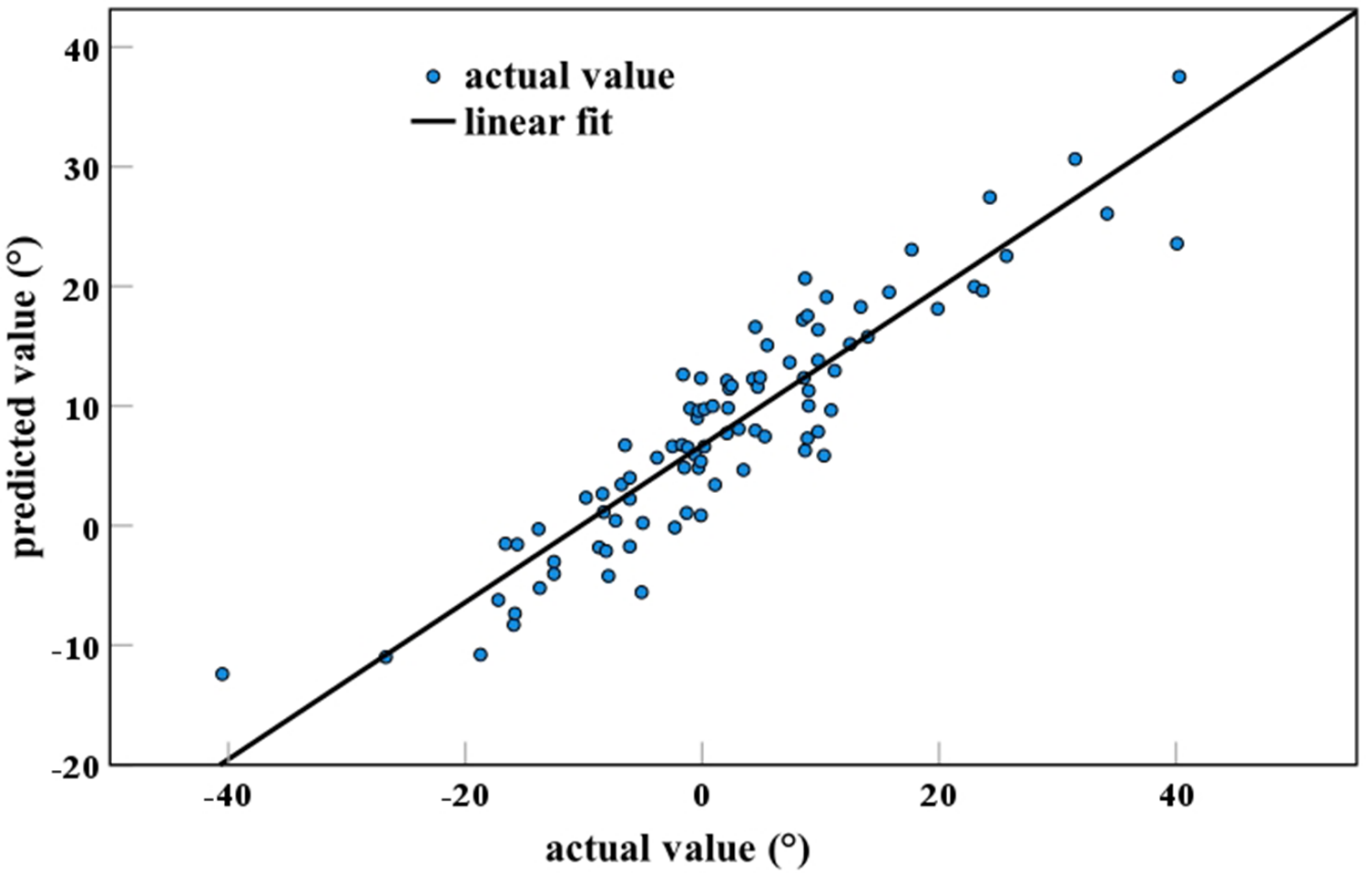
Validation of CL formula efficacy. The coefficient of determination equals 0.842.

This study validated the efficacy of the formula CL = 0.36 × OI−0.67 × OT−0.69 × T1S in AIS patients. There was a significant correlation (correlation coefficient = 0.917) between the actual measurements and the predicted values, with an R^2^ of 0.842 (Figure 7). R^2^ is the coefficient of determination, which represents the efficacy of the formula. The R^2^ value ranges from 0 to 1, and the closer it is to 1, the more effective it is.

## 4 Discussion

OI is a constant anatomical parameter in the cervical spine (19). Furthermore, there are differences in cervical sagittal alignment among various types of AIS (4). Before conducting our study, we hypothesized that there might be differences in OI among AIS patients with different curve types. The AIS patients in this study were classified into two groups: the main thoracic curve group and the main thoracolumbar/lumbar curve group. The results revealed a statistically significant difference in Cobb angle and OI between the two groups. We attribute this difference in OI to the varying effects of different scoliosis types on odontoid morphology. The difference in OI may be determined at birth, or it may arise from influences during the developmental process. We speculate that the possible influencing factors for this may include heredity, development, secondary ossification center, arterial supply, and hormones, all of which can affect odontoid morphology (25–29). The fusion of the secondary ossification center at the apex of the odontoid does not begin until adolescence (28). The development of odontoid morphology may be affected until the secondary ossification center is fully fused. In addition, there was also a difference in Cobb angle between the two groups, which suggests that the variations in Cobb angle may account for the difference in OI. The difference is consistent with the significant correlation between OI and Cobb angle.

Odontoid parameters include OI, OT, and C2S. Lee et al. conducted a study on odontoid parameters in healthy individuals, and Qin et al. conducted a study on odontoid parameters in patients with cervical spondylotic myelopathy. The results of the two studies consistently indicated significant correlations between odontoid parameters and several cervical sagittal parameters, and showed that OI can be used to assess cervical sagittal alignment (19, 20). In this study, the correlation analysis was conducted between odontoid parameters and other cervical sagittal parameters. The results showed significant correlations between odontoid parameters and other cervical sagittal parameters in AIS patients, as well as in AIS patients with different curve types. The results indicate that odontoid parameters could be used to describe cervical sagittal alignment in AIS patients with different curve types.

CL is a crucial parameter to assess cervical sagittal alignment. AIS patients often exhibit abnormalities in CL. The CL of AIS patients is significantly associated with neck and back pain, as well as cervical spondylosis (8, 18). The CL largely depends on both ends of the cervical spine. OI is the head influencing factor of CL, and T1S is the tail influencing factor. In theory, everyone has a CL that matches their OI and is optimal for them. A recent study in normal individuals demonstrated the following formula in determination of this: CL = 0.36 × OI−0.67 × OT−0.69 × T1S (19). The efficacy of the formula has been verified in normal populations (21). However, regarding the applicability of this formula to AIS patients, the efficacy thereof was verified in our study. This formula has the potential to serve as a reference standard to evaluate cervical sagittal alignment in AIS patients. It could be utilized to predict the optimal value of CL in AIS patients and evaluate the cervical sagittal alignment in AIS patients before and after surgery, or during the follow-up period. However, the formula has certain limitations, as it was developed based on a limited population with an older average age. Further studies can be conducted on younger and larger populations in order to develop a more accurate formula to predict CL.

AIS patients show abnormalities in both coronal and sagittal planes (30, 31). The sagittal alignment of AIS is receiving increasing attention. Several studies have indicated the presence of abnormal cervical sagittal alignment in AIS patients (4, 32, 33), and a previous study has shown correlations between cervical sagittal parameters and coronal plane deformities in AIS patients (13). Consistent with previous studies, our results showed significant correlations between OI and Cobb angle in AIS patients with a main thoracic curve.

There are limitations to our study. First, this study was conducted retrospectively at a single center, which could introduce bias. Second, the sample size was small, and a more detailed classification of scoliosis types could not be conducted for comparison. In the future, large-scale and multicenter studies should be conducted.

## 5 Conclusion

There may be a difference in OI between AIS patients with a main thoracic curve and those with a main thoracolumbar/lumbar curve. Odontoid parameters could be used to describe cervical sagittal alignment in AIS patients with different curve types. The formula CL = 0.36 × OI−0.67 × OT−0.69 × T1S could be used to predict the optimal CL in AIS patients.

## Data Availability

The raw data supporting the conclusions of this article will be made available by the authors, without undue reservation.
